# Ultrasonic modulation of brain glymphatic transport: from observations to theranostic applications

**DOI:** 10.20517/cdr.2025.223

**Published:** 2026-04-21

**Authors:** Wonseok Choi, Min-Hyeok Jang, Chulhong Kim, Eun-Yeong Park

**Affiliations:** ^1^Department of Biomedical Engineering and Medical Sciences, College of Medicine, The Catholic University of Korea, Seoul 06591, Republic of Korea.; ^2^Department of Plant Science, Seoul National University, Seoul 08826, Republic of Korea.; ^3^Departments of Convergence IT Engineering, Electrical Engineering, Mechanical Engineering, and Medical Science and Engineering, POSTECH-CATHOLIC Biomedical Engineering Institute, Medical Device Innovation Center, Pohang University of Science and Technology, Pohang 37673, Republic of Korea.; ^4^Department of Bio and Brain Engineering, Korea Advanced Institute of Science and Technology, Daejeon 34141, Republic of Korea.

**Keywords:** Glymphatic system, brain, ultrasound modulation, drug delivery

## Abstract

The glymphatic system in the brain controls the cerebrospinal fluid (CSF) circulation and metabolic waste clearance, which is crucial for understanding the mechanisms and therapeutic opportunities of various brain pathologies. With the rapidly growing interest in its relationship with neurodegenerative conditions, including Alzheimer’s disease, its underlying processes are still not fully understood and remain under active investigation. A representative finding is that the glymphatic flow is passively driven by factors such as vascular pulsation, and studies have been conducted to modulate the glymphatic system using external stimuli to enhance waste clearance or to leverage CSF pathways for delivering chemotherapeutic agents. Particularly, glymphatic flow modulation holds great potential for improving drug delivery to the brain via intrathecal administration as an alternative to conventional systemic delivery, which is restricted by the blood-brain barrier (BBB). This review focuses on ultrasound (US) techniques for glymphatic system modulation, with the aim of augmenting glymphatic flow and ultimately improving drug delivery for brain cancer therapy. Given the limited number of cancer-related studies in the field, we comprehensively review US-based glymphatic modulation research to date and identify their implications and future opportunities for brain cancer applications.

## INTRODUCTION

The “glymphatic” (“glia” plus “lymphatic”) system refers to the brain’s waste-clearance pathway, which encompasses periarterial cerebrospinal fluid (CSF) influx, CSF–interstitial fluid (ISF) exchange, perivenous CSF efflux, and final drainage through meningeal lymphatic vessels (mLV)^[1-4]^. CSF enters the brain parenchyma via periarterial spaces, driven primarily by arterial pulsation and respiratory dynamics^[5-8]^. It then exchanges with ISF through aquaporin-4 (AQP4) water channels located on astrocytic endfeet surrounding the cerebral vasculature^[9-13]^. This convective flux enables the removal of neurotoxic metabolites - including amyloid-β, tau, and lactate - from the interstitial compartment^[14-18]^. Cleared solutes subsequently migrate toward perivenous spaces and ultimately drain into the mLVs and systemic circulation^[19-21]^. Understanding the dynamics of glymphatic transport has therefore become central to studies of brain waste clearance and neurodegenerative disease mechanisms^[22-24]^.

Alongside this mechanistic understanding, there has been growing interest in exploiting the glymphatic system as a therapeutic delivery route for brain diseases^[25-27]^. Intrathecal administration enables therapeutics to enter the CSF, providing a complementary pathway to the classical blood-brain barrier (BBB) and potentially improving delivery efficiency by bypassing BBB filtration^[28-30]^. However, glymphatic flow is inherently passive, relying on convection generated by arterial pulsation, and its efficiency is substantially diminished in pathological conditions such as Alzheimer’s disease, Parkinson’s disease, traumatic brain injury, and cerebral small vessel disease. In brain tumors such as gliomas, reduced glymphatic transport has been reported, correlating with clinical manifestations including brain edema and intracranial hypertension^[31,32]^. Consequently, using the glymphatic pathway for chemotherapeutic delivery is paradoxically limited in many brain disorders.

**Figure 1:**
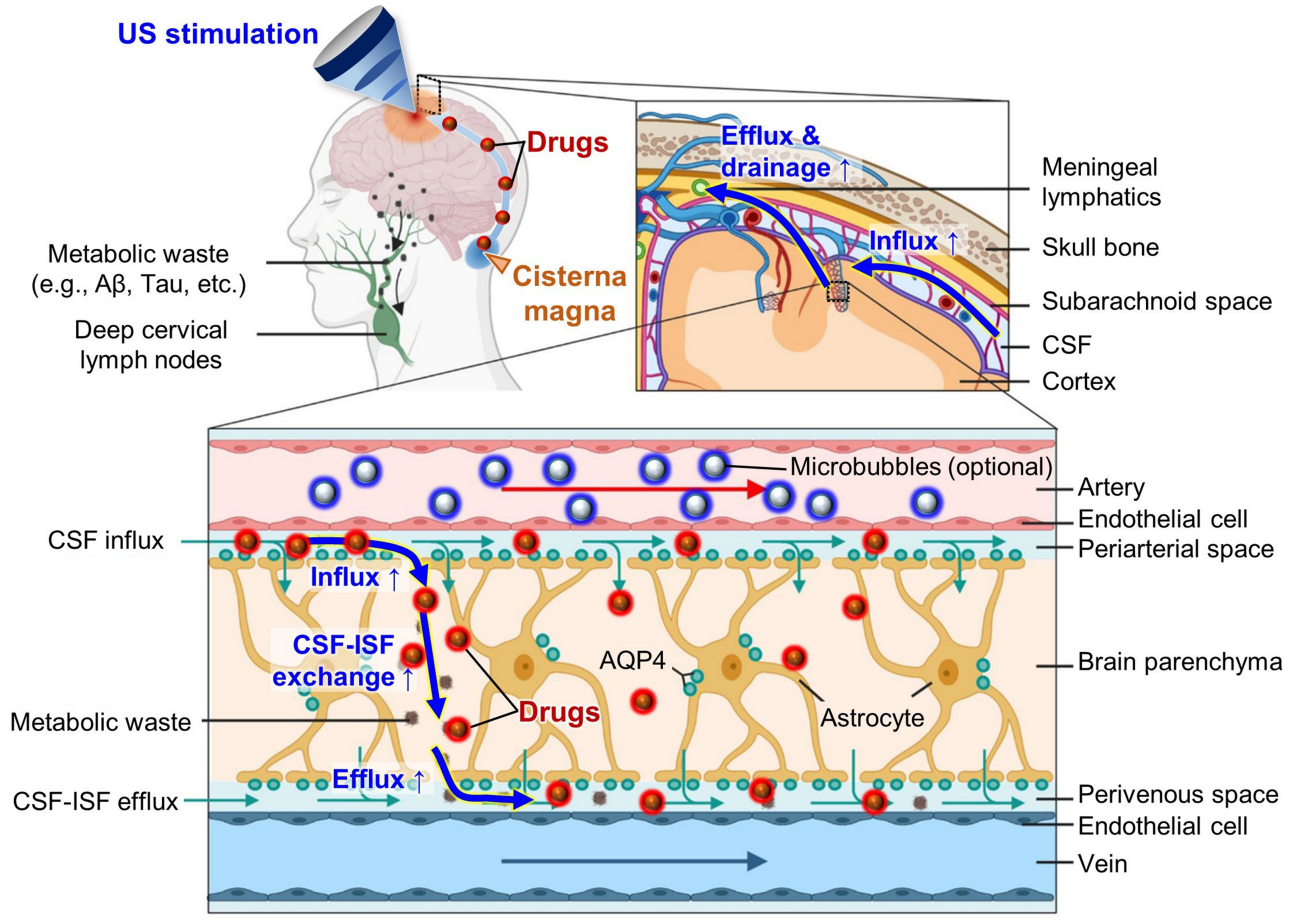
US-based augmentation of glymphatic flow for improved drug delivery via cisterna magna injection. Reproduced from Ref.^[33]^. Springer Nature under the CC BY 4.0 license. Copyright © 2023 The Authors. US: Ultrasound; Aβ: amyloid-β; CSF: cerebrospinal fluid; ISF: interstitial fluid; AQP4: aquaporin-4.

To achieve controlled upregulation of glymphatic flow, we review ultrasound (US)-based modulation studies that have demonstrated significant augmentation of glymphatic transport, highlighting its potential as an alternative drug-delivery pathway for brain diseases [Figure 1]. US modulation has been extensively studied for its efficacy in reversible BBB opening, owing to its non-invasiveness, non-ionizing nature, and capability for localized targeting^[34-37]^. Similar principles also apply to US-mediated glymphatic augmentation, which has been observed under clinically relevant exposure levels and across various imaging modalities, from live functional imaging to histological analysis^[38,39]^. While most glymphatic-focused studies have centered on neuro-degenerative diseases such as Alzheimer’s disease, research on brain tumors remains at an early stage^[31,32]^. Given the limited number of tumor-related reports, we broadly introduce US-based approaches for augmenting glymphatic flow and discuss their implications for improving drug delivery to brain tumors.

## THE GLYMPHATIC SYSTEM AS AN ALTERNATIVE DRUG DELIVERY ROUTE

Despite the severity of central nervous system diseases, including neurodegenerative disorders, stroke, and cancer, therapeutic drug delivery into the brain remains physiologically challenging. Systemic drug delivery is restricted by the BBB, which consists of tight junctions between vascular endothelial cells that block most exogenous molecules and permit only a small fraction of lipophilic, low-molecular-weight compounds (~0.4 kDa)^[40]^. As a result, only minimal amounts of systemically administered drugs reach the brain parenchyma, while most circulating drugs unintentionally accumulate in peripheral tissues. BBB-opening techniques such as focused US can reversibly widen endothelial junctions to increase drug penetration, but transport still relies on passive diffusion and remains limited by molecular size (approximately up to 70 kDa)^[41]^.

As a more direct route, drug delivery through the glymphatic system utilizes CSF influx into the perivascular space (PVS) surrounding arteries and subsequent CSF–ISF exchange through astrocytic AQP4 channels to enter the brain parenchyma^[42]^. Instead of intravenous injection for systemic delivery, glymphatic delivery requires invasive administration to the CSF, such as intrathecal injection into the cisterna magna. Although even large molecules can reach the PVS via glymphatic transport, penetration from the PVS into the parenchyma is constrained by the narrow extracellular gaps between astrocytes, which have been reported to be as small as ~20 nm^[43]^. Thus, depending on molecular size, CSF-delivered agents may efficiently enter the brain parenchyma or remain confined within periarterial spaces.

While delivery efficiency must be evaluated carefully, glymphatic transport at least bypasses the stringent filtering imposed by the BBB, offering more direct access to the PVS. Both routes remain size-limited, but perivascular astrocytic gaps may accommodate larger molecules than the BBB’s endothelial junctions, making the glymphatic pathway particularly attractive for macromolecular therapies. Moreover, glymphatic flow may naturally support brain-wide dispersion, which could be advantageous for certain therapeutic strategies. Conversely, for cancer applications requiring localized delivery, US-based focused augmentation of glymphatic flow may provide a feasible method to enhance drug penetration specifically at a targeted region of interest.

## THE GLYMPHATIC SYSTEM AND ITS IMPLICATION FOR BRAIN TUMORS

**Figure 2:**
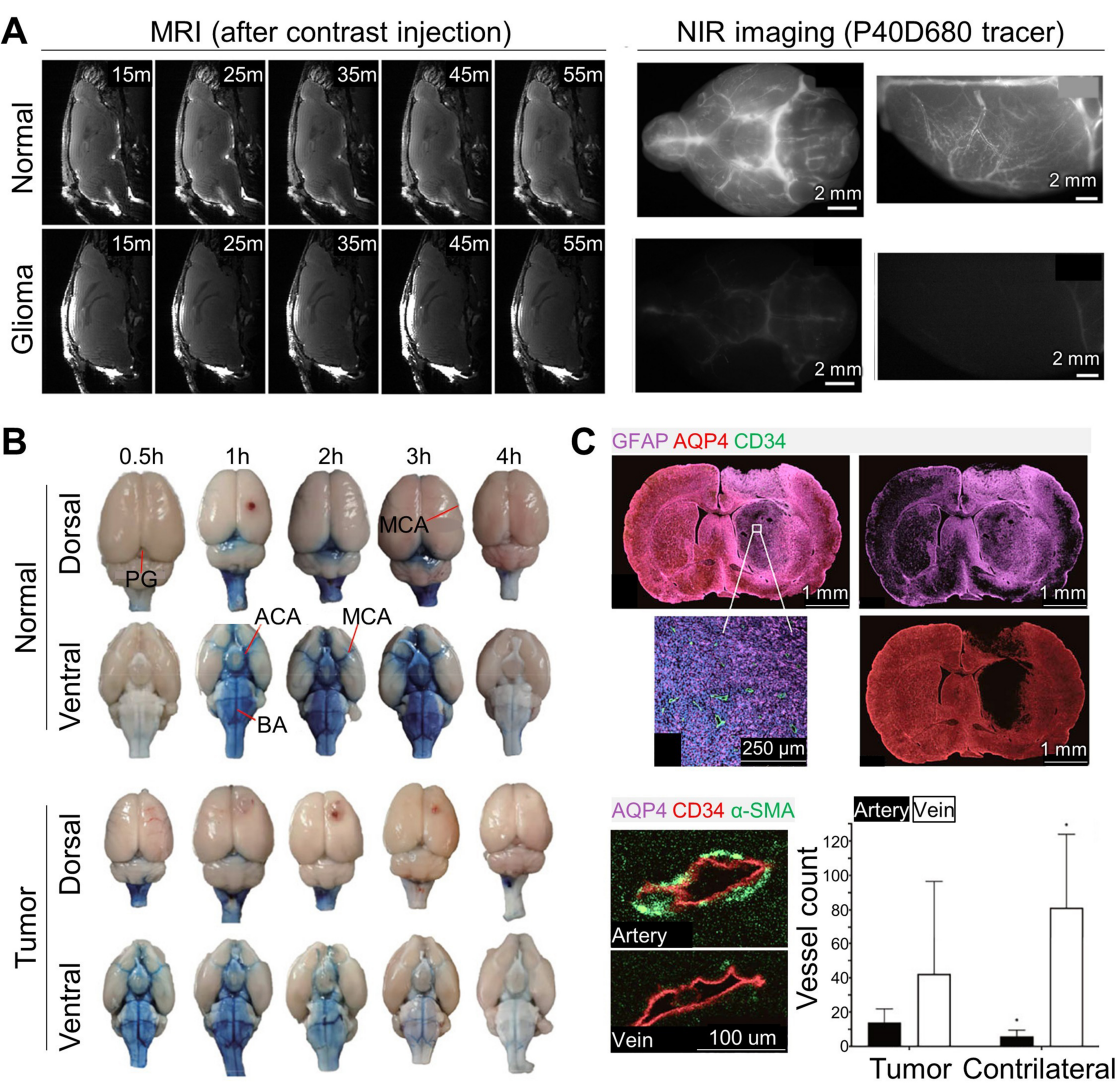
Glymphatic flow alterations in glioma. (A) Contrast-enhanced MRI images (*in vivo*) acquired 15, 25, 35, 45, and 55 min after cisterna magna injection, and NIR images (*ex vivo*) of the basal and dorsal regions using the P40D680 tracer, showing suppressed dispersion of contrast agents in glioma models; (B) Evans blue distribution in the dorsal and ventral sides of normal and tumor-bearing brains; (C) Histological images demonstrating the relationship between glymphatic impairment and AQP4 expression and vascular changes. (A) Reproduced from Springer Nature^[32]^ under the CC BY 4.0 license. Copyright © 2019 The Authors. (B and C) Reproduced from Springer Nature^[44]^ under the CC BY 4.0 license. Copyright © 2022 The Authors. MRI: Magnetic resonance imaging; NIR: near-infrared; AQP4: aquaporin-4; PG: pineal gland; MCA: middle cerebral artery; ACA: anterior cerebral artery; BA: basilar artery; GFAP: glial fibrillary acidic protein (astrocytic marker); CD34: cluster of designation 34 (vascular endothelial marker); α-SMA: alpha smooth muscle actin (vascular smooth-muscle/pericyte marker).

Ma *et al.* studied reduced CSF drainage in GL261 glioma-bearing mouse models using magnetic resonance imaging (MRI) and dynamic near-infrared (NIR) imaging^[32]^. The initial expectation was the opposite - that brain edema and intracranial hypertension in glioma patients would accelerate CSF drainage due to increased pressure. However, contrast-enhanced MRI and NIR imaging following cisterna magna injection showed that CSF circulation was markedly suppressed in glioma models, with tracer signal retained at the injection site rather than diffusing into the subarachnoid spaces or the basal regions around the Circle of Willis [Figure 2A]. CSF drainage into systemic blood circulation and cervical lymph nodes were substantially delayed and decreased, and perineural drainage along the optic nerves - observed using a macromolecular MRI tracer (Gadospin D, 17 kDa) - showed persistent tracer retention, indicating impaired drainage in glioma. Instead of normal glymphatic outflow, the study suggested that CSF drainage was redirected through the spinal cord in glioma.

Toh and Siow investigated alterations in glymphatic flow in glioma patients using the diffusion tensor imaging (DTI) analysis along the perivascular space (ALPS)^[31]^. ALPS reflects the diffusivity of perivascular CSF flow in the left-right direction, normalized by diffusivity along projection and association fibers, and is widely used to evaluate glymphatic function. This study analyzed 201 glioma patients (grades II-IV) by examining preoperative DTI-derived ALPS indices, isocitrate dehydrogenase 1 (*IDH1*) mutation status, and peritumoral edema or tumor volumes. The ALPS index was significantly lower in grade IV *vs.* grades II-III, and in *IDH1* wild-type *vs.* mutant gliomas, suggesting an association between impaired glymphatic flow and tumor aggressiveness. In addition, the ALPS index inversely correlated with peritumoral edema volume, indicating imbalanced CSF influx and efflux in gliomas.

Xu *et al.* analyzed factors contributing to reduced glymphatic outflow in glioma, which may limit the drug-delivery efficiency during intrathecal treatment^[44]^. Common glioma-related complications - such as brain edema and elevated intracranial pressure - may hinder therapeutic penetration into tumors, along with the restrictive BBB and blood-tumor barrier (BTB). Imbalanced CSF production and drainage may also contribute, due to reduced astrocytic AQP4 expression, a key regulator of water transport^[45-47]^. Their study used six-week-old male Sprague-Dawley rats injected with C6 glioma cells in the right striatum, measuring intracranial pressure, CSF flow dynamics via T1-weighted MRI, *ex vivo* glymphatic mapping using cisterna magna injection of Evans Blue, and histological imaging of AQP4 distribution. Intracranial pressure in the glioma group was nearly doubled, and MRI T1 signals were significantly lower in the tumor-bearing region. Evans Blue imaging confirmed globally reduced glymphatic transport, especially in the pineal recess and ventral regions near the Circle of Willis [Figure 2B]. Immunofluorescence showed reduced alpha-smooth muscle actin (α-SMA) expression and increased cluster of designation 34 (CD34)-positive vessels, indicating vascular immaturity. Reduced AQP4 and CD34^+^α-SMA^-^ veins were also observed in the tumor region, suggesting impaired CSF drainage [Figure 2C].

Given such observations, augmenting glymphatic flow may be a key strategy for overcoming the drug-delivery resistance of brain tumors^[48-50]^. Enhanced CSF drainage could alleviate intracranial hypertension and edema, thereby facilitating the transport of immune cells as well as exogenous chemotherapeutics into tumors. Furthermore, glymphatic-flow augmentation may provide new therapeutic opportunities for brain tumors, either by leveraging intrathecal administration to bypass BBB/BTB limitations or by improving CSF–ISF exchange dynamics.

## MODULATION METHODS FOR GLYMPHATIC FLOW AUGMENTATION

Various external stimulation modalities - optical, electromagnetic, and mechanical - have been reported to alter glymphatic flow. Optical approaches include visible sensory stimulation using frequency-modulated light flickering (~40 Hz) to induce gamma-wave activity, as well as transcranial NIR (tNIR) photostimulation, which directly illuminates brain tissue and facilitates astrocytic or neuronal activity^[51-54]^. Flickering-light stimulation can be delivered completely non-invasively using surrounding light-emitting diode (LED) sources during wakefulness, influencing multiple brain regions and enabling synergistic combination with other sensory modalities such as auditory stimulation^[51]^. Flicker stimulation modulates adenosine signaling, leading to augmented glymphatic flow through altered AQP4 polarization and enhanced vasomotion^[53]^. More directly, tNIR photostimulation uses light that penetrates the skin and skull - typically at wavelengths above 800 nm - to stimulate neural and glial function while minimizing thermal damage^[52]^. Among its various effects, tNIR has been shown to increase the contractility and pumping function of mLVs, thereby promoting ISF and CSF clearance from the brain^[52,54]^. In addition, tNIR induces local vasodilation and relaxation of blood vessels and mLVs through nitric oxide (NO) release, which further enhances glymphatic flow^[54]^.

Electromagnetic stimulation methods such as transcranial direct current stimulation (tDCS) and repetitive transcranial magnetic stimulation (rTMS) have also been shown to promote glymphatic transport^[55,56]^. These modalities have been clinically used as a complementary therapy for neurological disorders, including Alzheimer’s disease, aiding amyloid-β clearance. tDCS typically delivers ~2 mA of direct current between two scalp electrodes for several tens of minutes^[55]^ and has been shown to modulate astrocytic inositol trisphosphate (IP_3_)/Ca^2+^ signaling, thereby improving CSF–ISF exchange. Similarly, rTMS applies rapidly changing magnetic fields generated by a scalp-mounted coil, inducing electric currents in cortical tissue that augment glymphatic transport by modulating astrocytic reactivity, AQP4 polarization, and mLV dilation^[56]^.

Recent studies suggest that low-frequency vibration or subtle mechanical stimulation can promote CSF dynamics and potentially enhance mLVs function^[57,58]^. Observations that CSF movement is influenced by physiological oscillations - cardiac pulsation and respiration - raise the possibility that externally replicating these rhythms may facilitate lymphatic clearance. Vijayakrishnan Nair *et al.* used neck-region functional MRI (fMRI) to demonstrate that human CSF movement is simultaneously and synchronously driven by low-frequency (< 1 Hz) hemodynamic oscillations and respiration^[57]^. In a complementary animal study, Choi *et al.* showed that low-frequency (2 Hz) auricular vagus nerve stimulation (aVNS) increases arterial vasomotion and enhances CSF influx along the branches of the middle cerebral artery, demonstrating a noninvasive method to modulate intracranial fluid flow^[58]^.

Among these stimulation modalities, US offers a uniquely advantageous profile for glymphatic modulation because it delivers acoustic energy with high spatial precision, enabling targeted stimulation of deep brain structures that regulate CSF–ISF exchange^[59-61]^. Its mechanical effects - localized pressure oscillations and micro-vibrations of vessel walls - can directly enhance arterial pulsatility, a primary driver of glymphatic influx. Furthermore, low-intensity US combined with microbubbles can transiently and reversibly modulate BBB permeability, further influencing perivascular fluid movement^[62]^. Unlike optical, electrical, or magnetic stimulation, focused US can access specific vascular regions with millimeter-scale accuracy, and its stimulation parameters (e.g., frequency, pulse pattern, duty cycle) can be precisely tuned to optimize glymphatic clearance. These characteristics position US as a powerful and flexible tool for both mechanistic research and potential therapeutic augmentation of glymphatic function.

The different glymphatic flow modulation methods and their key characteristics are summarized in Table 1.

**Table 1:** Comparison table of glymphatic flow modulation methods

**Modulation method**	**Principle**	**Strengths**	**Limitations**
Sensory stimulation (visible, auditory)	Frequency-modulated stimulation (~40 Hz) to induce gamma-wave activity	Non-invasive, synergistic combination with other sensory stimuli	Mild effect and physiology-dependent, not localized
tNIR photostimulation	Direct illumination of brain tissue to facilitate astrocytic or neuronal activity	Localized stimulation	Invasive, limited tissue penetration
Electromagnetic stimulation (tDCS, rTMS)	Direct current or alternating magnetic field generated from the electrodes or coils on the scalp	Non-invasive, clinically established	Indirect, limited localization
Mechanical stimulation	Low-frequency vibration or subtle mechanical stimulation to mimic physiological oscillations	Non-invasive, direct enhancement of natural driving forces	Limited localization, difficult to quantify stimulation level
US stimulation	Direct insonication of brain tissue to bring localized or universal effects	Non-invasive, high spatial precision, deep tissue stimulation	Penetration and focusing affected by skull

tNIR: Transcranial near-infrared; tDCS: transcranial direct current stimulation; rTMS: repetitive transcranial magnetic stimulation; US: ultrasound.

## STUDIES ON US-MODULATED GLYMPHATIC TRANSPORT

Building on the unique strengths of US, recent studies have begun to characterize how US actively alters glymphatic transport. These investigations span diverse experimental frameworks, including MRI, optical imaging, and molecular assays, collectively demonstrating that US can enhance CSF influx, promote CSF–ISF exchange, and accelerate metabolic waste clearance. Importantly, several studies have further identified the biophysical and molecular pathways through which US exerts these effects.

Aryal *et al.* investigated US-induced enhancement of intrathecal drug delivery using both small- and large-molecule agents^[38]^. They focused on the problem of limited drug penetration into the brain parenchyma following intrathecal administration, despite its potential to bypass the BBB, which blocks more than 98% of systemically delivered therapeutics. Because glymphatic transport is largely passive and driven by arterial pulsation, they hypothesized that US combined with microbubbles could similarly modulate glymphatic flow from the CSF into PVSs and ultimately into the interstitial area. To transcranially insonify the entire rat brain, they applied low-frequency (650 kHz), low-intensity focused US at parameters within FDA-approved diagnostic limits (MI = 0.25, duty cycle = 7.7%, duration = 10 min). Using 3D T1-weighted MRI following intrathecal injection of a Gd-chelate contrast agent (1 kDa), they observed a significant augmentation of glymphatic transport (72%-101%), extending from periarterial regions into the parenchyma for up to 3 h post-US. To compare the transport of small *vs.* large molecules, they delivered Alexa Fluor 555-conjugated dextran-1 (~1.5 kDa) and Alexa Fluor 555-conjugated ABT-806 (~155 kDa) and analyzed parenchymal penetration via fluorescence microscopy. US insonification enabled significant penetration of the small-molecule dye into the parenchyma, whereas the large antibody conjugate accumulated primarily within PVSs. A key implication of this work is the demonstration that diagnostic-level, low-intensity US can meaningfully augment glymphatic-mediated drug delivery, supporting its translational potential in clinical settings. However, the observed size-dependent transport effects warrant further investigation, particularly regarding species-dependent anatomical factors and the need to evaluate a broader range of molecular sizes.

**Figure 3:**
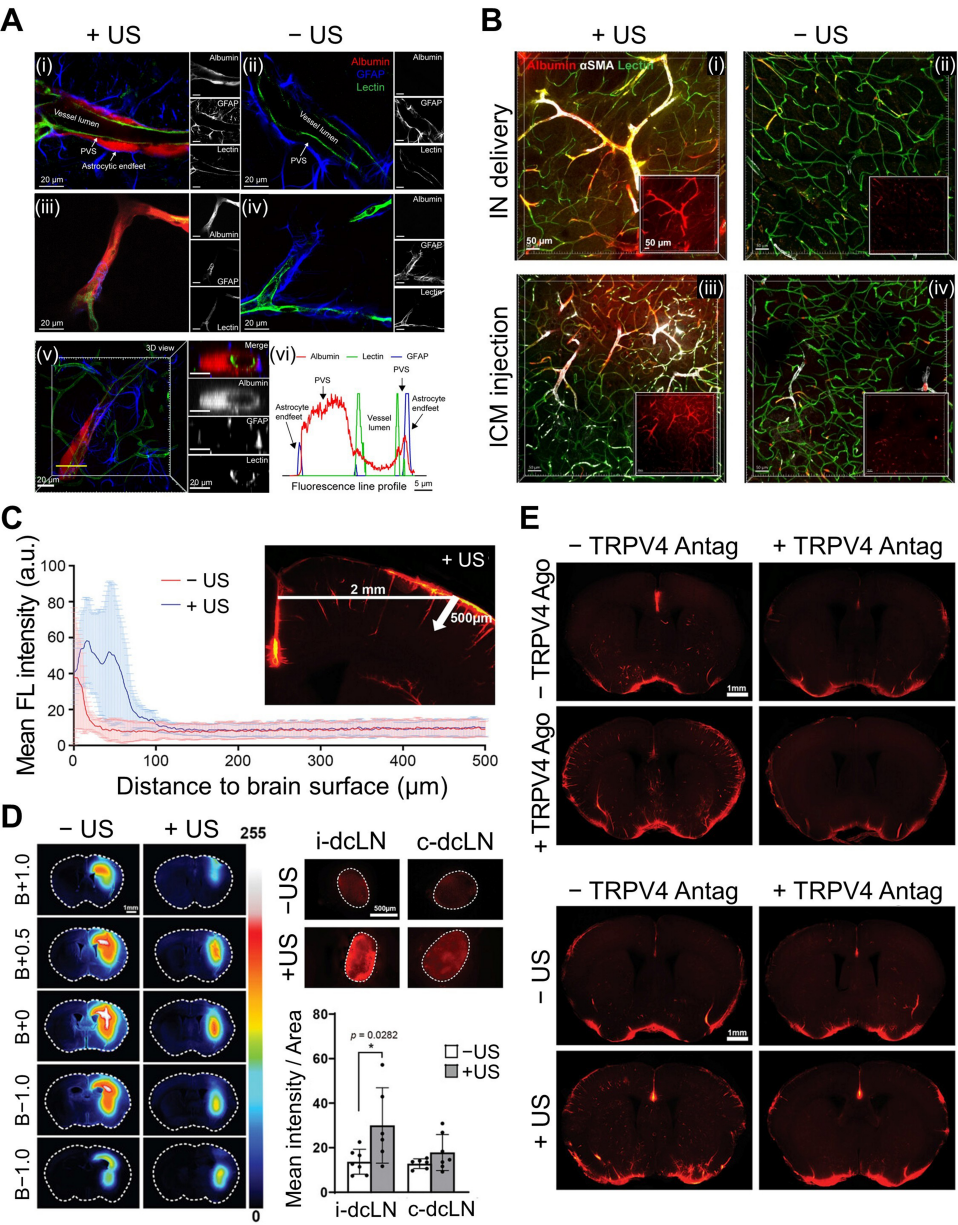
US-augmented glymphatic transport across influx pathways, vessel types, and clearance routes in mouse brain. (A) Representative confocal images of (i and ii) large and (iii and iv) small blood vessels with and without US-treatment (+US/-US), showing US-driven tracer movement along the PVS, (v and vi) Cross-sectional images and the corresponding fluorescence profiles confirming albumin tracer transport within the PVS; (B) Representative fluorescence images from (i and ii) the IN group and (iii and iv) the ICM group with and without US-treatment (+US/-US), demonstrating enhanced albumin penetration into the interstitial space; (C) Tracer penetration depth from the pial surface in coronal sections, demonstrating deeper tracer infiltration with US treatment; (D) Representative coronal brain sections at multiple bregma levels and corresponding dcLN images, showing reduced tracer residues in the parenchyma and increased dcLN accumulation following US treatment; (E) Representative images after pharmacological manipulation with TRPV4 agonist and/or antagonist, with and without US-treatment, illustrating TRPV4-dependent regulation of glymphatic influx and clearance. (A and B) Reproduced from Ye *et al.*^[63]^ with permission from the authors. Copyright © 2023 The Authors. (C-E) Reproduced from John Wiley and Sons^[64]^ under the CC BY 4.0 license. Copyright © 2024 The Authors. US: Ultrasound; PVS: perivascular space; IN: intranasal; ICM: intracisterna magna; dcLN: deep cervical lymph node; TRPV4: transient receptor potential vanilloid-4; GFAP: glial fibrillary acidic protein; αSMA: alpha smooth muscle actin; FL: fluorescence; B: bregma; i-dcLN: ipsilateral deep cervical lymph node; c-dcLN: contralateral deep cervical lymph node; Ago: agonist; Antag: antagonist.

Ye *et al.* further advanced mechanistic understanding by directly visualizing how US drives glymphatic transport at the microscopic level^[63]^. Using intranasal or cisterna magna administration of fluorescent albumin tracers followed by focused US with microbubbles, the authors performed optical tissue clearing and 3D confocal microscopy to reconstruct the CSF transport pathway. They revealed that US did not simply increase bulk tracer accumulation; instead, it amplified movement along the canonical glymphatic route–first into the PVS, then across astrocytic endfeet into the interstitial parenchyma [Figure 3A]. This stepwise enhancement confirmed that US facilitates physiological glymphatic flow rather than inducing nonspecific leakage. A key mechanistic insight emerged from their vessel-type analysis. Quantification across αSMA-positive arterioles *vs.* lectin-positive vessels demonstrated that US most strongly enhances influx along arterioles, followed by capillaries and then venules [Figure 3B], consistent with arterial pulsation being the primary natural driver of glymphatic transport. Moreover, US increased not only perivascular entry but also CSF–ISF exchange, enabling deeper tracer penetration into cortical tissue. Together, these findings support a model in which microbubble oscillations amplify vessel-wall motion, thereby strengthening each sequential step of glymphatic transport.

Beyond structural modulation, recent studies have shown that US can also regulate glymphatic transport through defined ion-channel-mediated pathways. Wu *et al.* demonstrated that low-intensity US enhances both CSF influx and interstitial efflux by activating the mechanosensitive channel transient receptor potential vanilloid-4 (TRPV4) in astrocytes^[64]^. Using a combination of *in vivo* transcranial fluorescence imaging, *ex vivo* optical clearing, and molecular assays, they visualized a marked increase in tracer entry into periarterial spaces and deeper cortical layers [Figure 3C] while simultaneously observing accelerated clearance toward the deep cervical lymph nodes (dcLNs) [Figure 3D]. At the cellular level, low-intensity US induced TRPV4-dependent Ca^2+^ influx, which activated calmodulin (CaM) and promoted AQP4 translocation to the astrocyte surface, thereby increasing water permeability along the glymphatic pathway. Pharmacological experiments using TRPV4 agonists and antagonists confirmed this mechanism: activating TRPV4 reproduced the US-induced augmentation, whereas blocking TRPV4 abolished both influx and clearance effects [Figure 3E]. The study further demonstrated improved amyloid-β clearance, showing reduced residues at the injection site and increased accumulation in dcLNs, again in a TRPV4-dependent manner. High-resolution confocal imaging revealed transient astrocyte swelling (20-40 min) due to increased AQP4-mediated water influx, followed by complete recovery by 65 min, indicating a reversible and non-injurious physiological response. Overall, the results demonstrate that low-intensity US augments glymphatic transport through a TRPV4-CaM-AQP4 water-transport mechanism, supporting both CSF influx and interstitial clearance.

In contrast to studies relying primarily on *ex vivo* imaging or MRI-based snapshots, Gong *et al.* directly visualized the moment US was applied, using a custom two-photon imaging platform built around a ring-shaped transducer that allowed US delivery and optical imaging simultaneously^[39]^. This configuration enabled real-time observation of how microbubble-assisted sonication deforms vessel walls and alters glymphatic transport in the living mouse brain. Using a cranial window preparation and a dual-modality setup, the authors simultaneously tracked CSF tracer movement and vessel wall behavior during and after US exposure. This approach revealed that US induced rapid, cyclic vessel deformation, characterized by alternating invagination and dilation synchronized with sonication. These mechanical changes were substantially larger than in sham conditions, with a 7.25-fold greater diameter change and a 3.09-fold faster deformation rate. Concurrently, the perivascular tracer signal exhibited a marked decline, indicating accelerated local clearance during US exposure. Quantitatively, tracer intensity decreased 1.86-fold more than in controls, and the maximum clearance rate increased 4.57-fold, demonstrating that US enhances not only CSF influx pathways but also downstream efflux dynamics. A strong correlation between the rate of vessel deformation and the rate of tracer clearance (R^2^ = 0.82) further established a direct biophysical coupling between US-driven vascular motion and glymphatic transport efficiency. By classifying vessels as small (10-40 μm), medium (40-70 μm), or large (70-100 μm), the study also showed that while small vessels exhibited the greatest absolute deformation, larger vessels generated much stronger clearance responses. These findings indicate that US can modulate glymphatic flow across multiple vascular scales and that efflux along larger draining vessels may be particularly sensitive to US-induced mechanical forces.

**Figure 4:**
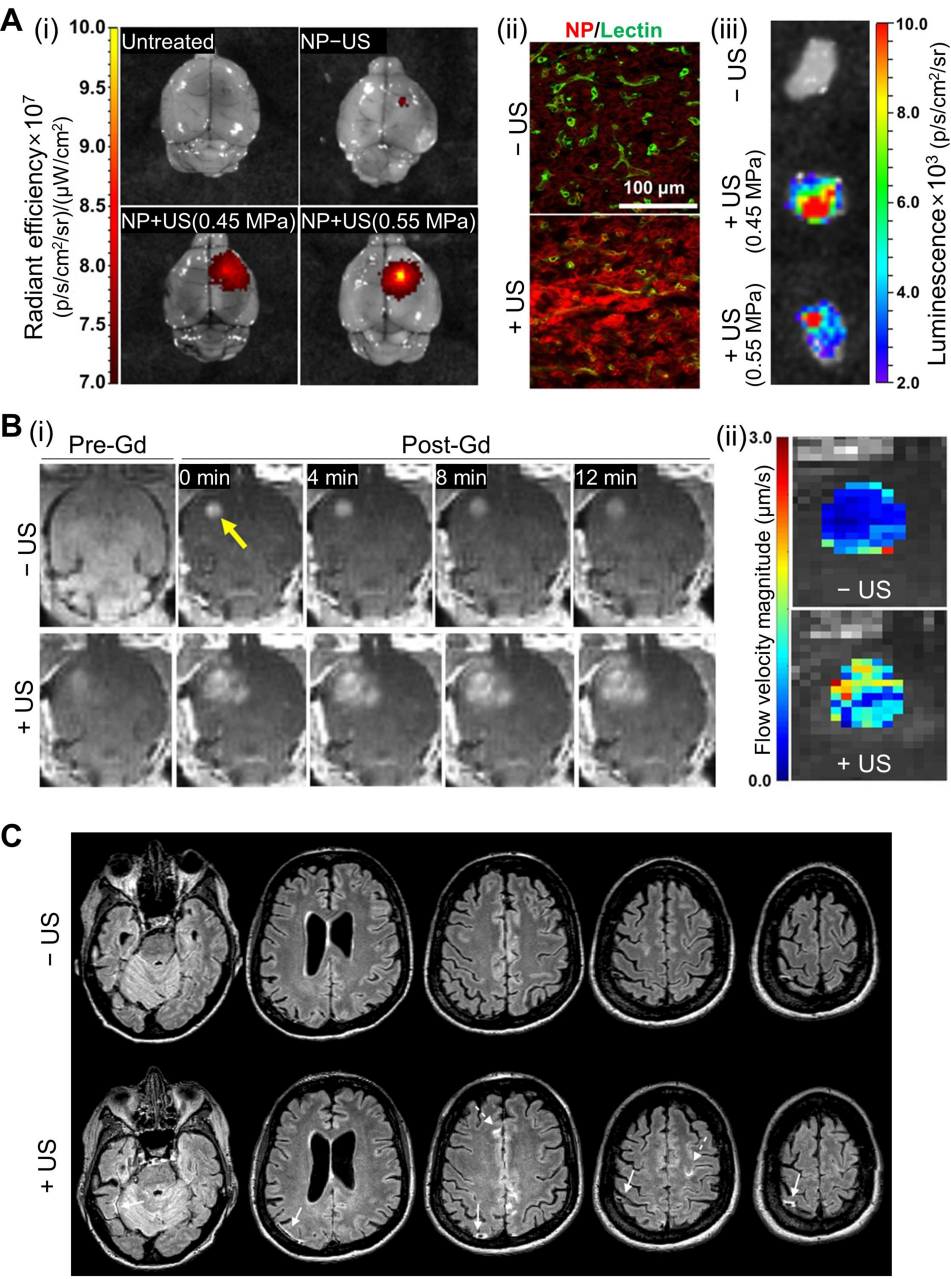
US-modulated glymphatic transport in disease models. (A) Enhanced influx and NP delivery in glioma-bearing mice. (i) Increased tumor uptake of fluorescent NPs following US with microbubble treatment. (ii) Confocal fluorescence imaging confirming deeper intratumoral penetration. (iii) Bioluminescence imaging showing elevated transgene expression after US-mediated BBB opening; (B) MRI-based assessment of BTB opening and interstitial flow changes. (i) Contrast-enhanced MRI with and without US-treatment demonstrating BTB permeability increase. (ii) Flow-velocity magnitude maps showing US-induced acceleration of interstitial transport; (C) Representative human FLAIR MRI showing sheath-like contrast enhancement around large cortical veins following MR-guided focused US-induced BBB opening in an AD patient. Subarachnoid contrast accumulation appears adjacent to the sonicated region, demonstrating a reversible BBB permeability response in human subjects. (A and B) Reproduced from Curley *et al.*^[65]^, with permission from AAAS. (C) Reproduced from Meng *et al.*^[66]^, with permission from John Wiley and Sons. Copyright © 2019 John Wiley and Sons. US: Ultrasound; NP: nanoparticle; BBB: blood-brain barrier; BTB: blood-tumor barrier; MRI: magnetic resonance imaging; FLAIR: fluid-attenuated inversion recovery; MR: magnetic resonance; AD: Alzheimer disease; Gd: gadolinium.

Curley *et al.* investigated how microbubble-assisted focused US can enhance ISF transport within brain tumors, thereby improving the delivery of locally and systemically administered therapeutics^[65]^. Although this work does not target glymphatic transport from the CSF, it is noteworthy because interstitial flow enhancement represents a complementary mechanism by which US can facilitate solute movement from perivascular pathways into tumor parenchyma. This study focuses on both the BTB and BBB, which together restrict the penetration of gene-therapy vectors and other macromolecular agents. The BTB limits convection from blood to tumor tissue due to elevated ISF pressure, whereas the BBB becomes relevant when infiltrating tumor cells extend beyond the tumor core. While microbubble-assisted focused US is already known to transiently and reversibly open both barriers, Curley *et al.* further evaluated its impact in glioma disease models by examining nanoparticle delivery and functional gene expression following US with microbubbles [Figure 4A]^[65]^. Specifically, fluorescence imaging of whole brains and excised tumors demonstrated increased tumor uptake of intravenously administered nanoparticles after focused US treatment [Figure 4A(i)]. To examine whether enhanced accumulation was accompanied by improved spatial distribution within tumors, confocal fluorescence imaging was performed, revealing deeper intratumoral penetration of nanoparticles relative to tumor microvessels after US treatment [Figure 4A(ii)]. To evaluate whether enhanced interstitial flow could also improve gene-vector delivery, the authors intravenously injected luciferase-bearing nanoparticles immediately before US treatment and measured tumor transgene expression using *ex vivo* bioluminescence imaging. Focused-US with microbubble resulted in ~4-fold increases in both total photon flux and average radiance compared with nanoparticles alone [Figure 4A(iii)], demonstrating that US enhances not only tracer dispersion but also functional gene-expression efficiency. They also demonstrated how focused US modulates interstitial transport by analyzing time-series contrast-enhanced MRI data [Figure 4B(i)]. Using an MRI-guided focused US system (3T MRI integrated with a 1.1 MHz spherical transducer and passive cavitation hydrophone), they selected eight treatment points per tumor and applied 0.45-0.55 MPa peak negative pressure for 2 min in 0.5% duty cycle. After BBB/BTB opening, serial T1-weighted MRI acquisitions with intravenously injected Gd-chelate (~0.75 nm) enabled voxel-wise diffusion modeling. In both U87 glioma and intracranial B16F1ova melanoma models, interstitial flow velocity significantly increased after US treatment, indicating US-driven augmentation of solute transport within tumor tissue [Figure 4B(ii)]. Finally, to assess transport of larger therapeutic carriers, the authors examined nanoparticles originally termed “brain-penetrating nanoparticles (BPNs)” - ~50-nm particles engineered to disperse efficiently through tumor interstitial spaces after local infusion. U87mCherry-bearing mice received systemic administration of BPNs carrying a ZsGreen reporter gene, followed by focused US insonification. Histological analysis showed a two-fold increase in transfection volume, demonstrating the feasibility that US-based interstitial-flow upregulation can improve the delivery of large-molecule drugs into brain tumors.

Meng *et al.* extended US-modulated glymphatic research into humans by examining contrast transport patterns following MR-guided focused US BBB opening in subjects with Alzheimer’s disease and amyotrophic lateral sclerosis^[66]^. In all 12 participants, reversible BBB opening was successfully achieved, and a subset of subjects exhibited characteristic perivenous and subarachnoid contrast enhancement on fluid-attenuated inversion recovery (FLAIR) MRI immediately after sonication [Figure 4C]. These hyperintensities appeared as sheath-like signal encasement along large cortical draining veins and branching enhancement within adjacent subarachnoid spaces, closely resembling glymphatic efflux routes previously identified in rodents. Notably, these patterns consistently reappeared across repeated treatments yet resolved within 24 h without clinical symptoms, suggesting a transient and physiologically driven response rather than tissue injury. Collectively, these observations provide the first radiologic evidence in humans that focused US may modulate glymphatic drainage along perivenous routes, supporting its translational potential for enhancing solute clearance in neurodegenerative disease.

A summary of the *in vivo* and *ex vivo* studies, including protocols, US parameters, and key findings, is provided in Table 2.

**Table 2:** Summary of *in vivo* and *ex vivo* US-modulated glymphatic transport studies

**Ref.**	**Model**	**Protocol**	**US parameters**	**Observation**
Aryal *et al.*^[38]^	• Rats (male, Long-Evans) • Normal	Anesthetized (isoflurane) › ICM inj. of MRI contrast agent › Pre-US MRI › Transcranial US (hair removed; whole brain) › Post-US MRI (up to 4 h p.i.; 7T, T1w)	[MB] - None [Transducer] • Focused • Fc: 650 kHz • FWHM: 2.78 × 12 mm [Sonication] • MI: 0.25 • PNP: 0.2 MPa • Duty cycle: 7.7% • Duration:10 min • Scan area: 8 × 10 mm	• Increased uptake into brain parenchyma by 72%-101% • Increased residual tracer at 3 h p.i.
		Anesthetized (isoflurane) › ICM inj. of FL-labeled agents (Alexa Fluor 555) › Transcranial US (hair removed; whole brain) › *Ex vivo* FL micro. (euthanized, tissue fixed, frozen-sectioned)		[FL dye: ~1.5 kDa] • Higher concentration in brain parenchyma at 1 h p.i. [FL-conjugated antibody: ~155 kDa] • Increased influx into PVS (peak at 1 h p.i.) • No influx into brain parenchyma
Meng *et al.*^[66]^	• Human • AD, ALS	IV inj. of MBs › US treatment › IV inj. of MRI contrast agent › MRI (0, 24 h p.t.; 3T, T1w/FLAIR)	[MB] • Commercial [Transducer] • Fc: 220 kHz	• Reversible BBB opening in all cases • Enhanced contrast in SAS adjacent to sonicated regions
Ye *et al.*^[63]^	• Mice (female, NIH Swiss) • Normal	Anesthetized (isoflurane) › IN inst. of FL-labeled tracer (Alexa Fluor 555; 24 μL) › IV inj. of MB (30 min p.inst.) › US treatment (hair removed; one side of thalamus) › IV inj. of lectin › *Ex vivo* FL imaging (euthanized at 15 min p.t., perfused, brain sectioned) › *Ex vivo* confocal micro. (optically cleared, immuno-stained: lectin/αSMA/GFAP)	[MB] • Commercial • Dose: 10 μL/kg [Transducer] • Focused • Fc: 1.5 MHz [Sonication] • P: 0.4 MPa • Pulse length: 6.7 ms • PRF: 5 Hz • Duration: 60 s	• Increased tracer accumulation (1.49× higher FL signal) • Enhanced tracer transport in PVS between vessel wall and astrocytic endfeet • Arteriole-dominant distribution: 8.75×/5.86× higher FL signal in arterioles/capillaries, with no significant difference in venules • Increased influx from PVS to IS
		Only changes: › ICM inj. of tracer (5 μL)		• Increased tracer accumulation (1.74× higher FL) • Arteriole-dominant distribution: 3.49×/2.16×/2.21× higher FL in arterioles/capillaries/venules
Wu *et al.*^[64]^	• Mice (C57BL/6JNarl) • Normal	Anesthetized (Zoletil + xylazine) › Drug administration (TRPV4 agonist/antagonist, AQP4 inhibitor, CaM inhibitor) › ICM inj. of FL-labeled tracer (Alexa Fluor 555; 6 μL) › US treatment › *Ex vivo* FL micro. (euthanized at 30 min p.i., brain sectioned and optically cleared) › *Ex vivo* confocal micro. (immuno-stained: TRPV4-Alexa Fluor 488, AQP4/GFAP-Alexa Fluor 633)	[MB] • None [Transducer] • Unfocused • Fc: 1 MHz [Sonication] • PRF: 1 kHz • Duty cycle: 1% • Duration: 5 min • I_SPTA_: 3.68 mW·cm^2^ (~98 kPa)	• Increased CSF tracer influx into PVS • Deeper and greater penetration into cortical parenchyma • Enhanced rostro-caudal distribution (bregma -2 to +1 mm)
				• Enhanced influx mediated by the TRPV4-CaM-AQP4 pathway • Transient astrocyte swelling due to water influx via AQP4 (20-40 min p.t.) • Full recovery of astrocyte volume (65 min p.t.)
		Anesthetized (Zoletil + xylazine) › ICM inj. of FL-labeled tracer (Alexa Fluor 555; 6 μL) › US treatment › *In vivo* FL imaging (0-64 min p.i.; skin incised; dorsal calvarium)		• Increased CSF tracer influx into PVS at 15 min • Greater tracer infiltration into brain parenchyma
		Anesthetized (Zoletil + xylazine) › Drug administration (TRPV4 agonist/antagonist) › Parenchymal inj. of FL-labeled tracer/human Aβ (Alexa/HiLyte Fluor 555; 12 μL; left striatum) › US treatment › *Ex vivo* FL micro. (3 h p.i.; euthanized, brain sectioned and dcLNs harvested; both optically cleared) › Tissue homogenization		• Reduced residual tracer/Aβ in the parenchymal injection site • Increased tracer/Aβ drainage to dcLNs • Enhanced waste clearance via a TRPV4-dependent pathway
Gong *et al.*^[39]^	• Mice (female, claudin-5-GFP transgenic, C57BL/6J background) • Normal	[Preparation: 2-3 weeks before US treatment] › Cranial window implantation (scalp incised, skull removed, brain covered with agarose, polyester film sealed with epoxy/dental cement) [Protocol] Anesthetized (ketamine + xylazine) › ICM inj. of FL-labeled tracer (Alexa Fluor 555; 10 μL) › IV inj. of MBs › US treatment (30 min p.i. of tracer) › Live *in vivo* two-photon micro.	[MB] • Commercial • Size: 1.34 ± 0.28 μm [Transducer] • Ring-type • Fc: 0.5 MHz • FWHM: 2 × 10 mm [Sonication] • PNP: 0.3 MPa • 1,000 cycles • PRF 5 Hz • Duration: 2 min	• Synchronized vessel deformation with US sonication (7.25× higher change in diameter) • Enhanced local clearance of injected CSF tracer (1.86× higher intensity, 4.57× faster clearance rate) • Significant linear correlation between vessel deformation and tracer clearance (R^2^ = 0.82) • Stronger clearance response in larger vessels: linear regression slopes of 2.61/0.85/0.24 for large (70-100 μm)/medium (40-70 μm)/small (10-40 μm) vessels
Curley *et al.*^[65]^	• Mice (male, athymic, nude) • Glioma (IC, U87)	Anesthetized (ketamine + dexdomitor) › IV inj. of FL-labeled NPs (Cy5; 1 μg/g) & MBs › US treatment (5 d p.t.i.) › *Ex vivo* FL imaging (6 h p.t.; euthanized, brain harvested, tumor dissected) › *Ex vivo* confocal micro.	[MB] • Albumin-shelled • 1 × 10^5^ per gram body weight [Transducer] • Focused • Fc: 1.1 MHz [Sonication] • PNP: 0.45/0.55 MPa in water • Duty cycle: 0.5% (10 ms pulses, 2 s interval) • Eight sonication spots	• Increased NP delivery across BTB/BBB • Whole-brain: 3.5×/4.5× higher FL signal at 0.45/0.55 MPa • Dissected tumor: 2.3× higher FL signal at 0.55 MPa; no significant increase at 0.45 MPa • Increased NP delivery into tumor microvessels and tissue
	• Mice (male, athymic, nude) • Glioma (IC, U87-mCherry) • Melanoma (IC, B16-F1-OVA)	Anesthetized (ketamine + dexdomitor) › IV inj. of luciferase-bearing NPs (1 μg/g) & MBs › US treatment (5 d/7 d p.t.i. for glioma/melanoma) › *Ex vivo* BL imaging (3 d p.t.; IP inj. of luciferin, euthanized at 5 min p.i., tumor harvested)		• Enhanced transgene expression in both tumor models: ~4× increases in total flux and average radiance
		Anesthetized (ketamine + dexdomitor) › IV inj. of MRI contrast agent (50 μL) & MBs › Pre-US MRI › US treatment (5 d/7 d p.t.i. for glioma/melanoma) › Re-inj. of MRI contrast agent › Post-US MRI (0, 4, 8, 12 min p.t.; 3T, T1w)		• Pre-US MRI: tumor visible via contrast-agent leakage from tumor vessels • Post-US MRI: increased contrast enhancement, indicating BBB/BTB disruption • Flow changes: ~2× increase in velocity magnitude; marked change in direction; increased convection transport
	• Mice (male, athymic, nude) • Glioma (IC, U87-mCherry)	Anesthetized (ketamine + dexdomitor) › IV inj. of MBs › US treatment (16 d p.t.i.) › CED inj. of ZsGreen-NPs (19 μg/20 μL; 0.33 μL/min) › *Ex vivo* confocal micro. (2 d p.i.; euthanized, perfused, brain harvested, frozen-sectioned)	Only changes: [Transducer] • Fc: 1 MHz [Sonication] • PNP: 0.45 MPa • Duration: 2 min	• ~2× increased transfection volume in tumor

US: Ultrasound; ICM: intracisterna magna; inj.: injection; MRI: magnetic resonance imaging; h: hour; p.i.: post-injection; T1w: T1-weighted; FL: fluorescence; micro.: microscopy; MB: microbubble; Fc: central frequency; FWHM: full-width at half-maximum; MI: mechanical index; PNP: peak negative pressure; min: minute; PVS: perivascular space; AD: Alzheimer disease; ALS: amyotrophic lateral sclerosis; IV: intravenous; p.t.: post-treatment; FLAIR: fluid-attenuated inversion recovery; BBB: blood-brain barrier; SAS: subarachnoid space; IN: intranasal; inst.: instillation; p.inst.: post-instillation; αSMA: alpha smooth muscle actin; GFAP: glial fibrillary acidic protein; P: pressure; PRF: pulse repetition frequency; IS: interstitial space; TRPV4: transient receptor potential vanilloid-4; AQP4: aquaporin-4; CaM: calmodulin; dcLNs: deep cervical lymph nodes; I_SPTA_: spatial-peak temporal-average intensity; CSF: cerebrospinal fluid; GFP: green fluorescence protein; IC: intracranial; NPs: nanoparticles; d: day; p.t.i.: post tumor implantation; BL: bioluminescence; IP: intraperitoneal; BTB: blood-tumor barrier; CED: convection-enhanced delivery.

## DISCUSSION AND FUTURE DIRECTIONS

Despite the substantial interest in the glymphatic system in the context of neurodegenerative diseases, only a limited number of studies have examined its role in brain cancers. Conventional glymphatic research has focused on the drainage and removal of metabolic waste in non-cancerous brain diseases, because these processes are directly related to alleviating symptoms and slowing disease progression. In contrast, research in brain cancer has traditionally emphasized angiogenic remodeling, immune interactions, and tumor-specific molecular signaling, leaving the contribution of glymphatic transport poorly defined. With respect to drug delivery, BBB opening has been the dominant strategy, and the idea of leveraging the glymphatic system as an alternative therapeutic route has only recently emerged. As summarized in the preceding sections, foundational studies characterizing glymphatic impairment in tumors may help clarify mechanisms of drug delivery resistance and motivate investigations into glymphatic flow augmentation as a complementary therapeutic strategy. Because US has already been shown to achieve safe, reversible BBB opening, its potential to simultaneously modulate glymphatic transport makes it a compelling candidate for synergistic enhancement of intrathecally delivered therapeutics, particularly when considering molecular size constraints and the extent of dispersion into interstitial or perivascular compartments.

At present, however, the available human evidence for US-mediated glymphatic modulation remains limited. Clinical MRI observations to date are derived from relatively small cohorts of patients with neurodegenerative diseases, and direct evidence in human brain tumor populations is currently lacking. Accordingly, the translational relevance of glymphatic augmentation for oncologic drug delivery should be interpreted with caution, and future clinical studies specifically designed for tumor-bearing patients will be required to establish its therapeutic potential in cancer.

Among various modulation modalities, US is uniquely capable of delivering focused energy to highly localized regions within the brain. This feature is advantageous for minimizing off-target toxicity in surrounding tissues - an especially critical consideration in oncology. However, most glymphatic modulation studies to date have been designed to influence broad cortical or subcortical regions simultaneously, and thus the potential benefit of localized glymphatic modulation remains underexplored. This raises an important question: which brain pathologies, beyond tumors, exhibit spatially localized glymphatic impairment, and to what extent can localized modulation improve therapeutic outcomes? Addressing this question may broaden the clinical relevance of US-based glymphatic modulation to conditions such as traumatic brain injury, stroke, or focal epileptic lesions, although these applications have not yet been systematically evaluated to the best of our knowledge.

At the same time, several key challenges remain for the effective translation of US-based glymphatic modulation. These include the lack of standardized US parameters across studies, incomplete understanding of dose-response relationships between acoustic exposure and glymphatic transport, and limited ability to noninvasively and quantitatively monitor glymphatic modulation in real-time. In transcranial applications, skull-induced acoustic attenuation, phase aberration, and focal distortion further complicate accurate energy delivery, potentially limiting penetration depth and spatial precision of US-based glymphatic modulation. In addition, disease-specific variability in vascular integrity, interstitial pressure, and perivascular architecture–together with inter-individual differences in skull thickness and geometry–may further complicate parameter optimization, particularly in heterogeneous tumor environments. Addressing these technical and biological challenges will be critical for advancing US-based glymphatic modulation toward reliable and reproducible clinical applications.

Observation of the glymphatic flow alterations has primarily relied on MRI and optical imaging, both of which enable dynamic visualization of contrast transport at different spatial scales. Contrast-enhanced MRI provides whole-brain, macroscale characterization of glymphatic pathways in both small animals and humans, whereas optical imaging allows microscale resolution in superficial regions of rodent brains through optical windows. An emerging complementary approach is photoacoustic imaging (PAI), which detects optically absorptive chromophores with ultrasonic resolution^[67,68]^. Because PAI can be deployed across a wide range of imaging depths - from photoacoustic microscopy to photoacoustic tomography - it effectively bridges the resolution-depth gap between pure optical imaging and tomographic modalities such as MRI or PET^[69-71]^. Although its application to glymphatic imaging is still recent^[72]^, PAI’s scalability and compatibility with US-based modulation systems suggest that it could become a valuable tool for monitoring US-augmented glymphatic flow^[73-75]^. Moreover, functional vessel-imaging methods such as ultrafast US Doppler could help identify pulsatile vascular segments and clarify their mechanistic relationship with glymphatic flow^[76-78]^.

Accurate assessment of glymphatic function, however, requires careful attention to experimental factors that directly influence lymphatic and glymphatic dynamics^[79]^. Bouta *et al.* systematically evaluated how key physical variables - animal posture, contrast agent injection volume, and mechanical tissue support - alter lymphatic contraction physiology^[80]^. Rather than treating these variables as procedural details, they should be recognized as critical control parameters that shape the reproducibility and interpretability of lymphatic imaging studies. First, mouse posture relative to gravity significantly affected lymphatic contraction frequency: upright positioning preserved normal contraction rates, whereas the supine position markedly reduced them, despite unchanged ejection fraction (EF). This finding implies that gravitational forces impose resistance that alters lymphatic pumping rhythms, a consideration especially relevant when imaging contrast transport toward mLVs. Second, injection volume had a dose-dependent inhibitory effect on contraction frequency; volumes of 4, 10, and 20 μL showed progressively reduced activity, with 20 μL producing the lowest frequency. Excessive volume may elevate intraluminal pressure or shear stress, triggering negative feedback mechanisms that suppress lymphatic contractions and potentially diverting tracer flow toward low-resistance pathways. Thus, minimizing injection volume to the effective threshold is essential for experimental consistency. Third, removal of skin and the resulting loss of external mechanical support significantly impaired lymphatic contraction. As contraction frequency decreased following skin removal, the authors suggested that external confinement contributes to maintaining physiological lymphatic pumping. Moreover, anesthesia has been shown to adversely affect glymphatic function and reduce transport toward the mLV^[81]^, underscoring the need for protocols that incorporate adequate wake periods or standardized anesthetic depth. Collectively, these observations highlight the importance of methodological consistency, transparent reporting, and careful control of experimental variables in studies evaluating glymphatic and lymphatic transport.

Despite these advances, several limitations remain. Most studies have been conducted in rodents with normal brain physiology, and the extent to which tumor-associated abnormalities - elevated interstitial pressure, vessel immaturity, heterogeneous extracellular matrix, or altered AQP4 localization - modulate US responsiveness is not yet known. Moreover, current findings on molecular-size dependence suggest that while US can enhance delivery of small agents via glymphatic pathways, the transport of large biological therapeutics may require additional strategies or optimized modulation paradigms.

Looking forward, the convergence of US neuromodulation, lymphatic biology, and intrathecal drug delivery opens several compelling research directions. Future work is needed to define optimal US parameters for tumor-bearing brains, evaluate therapeutic distribution under pathological glymphatic impairment, and determine how localized *vs.* global glymphatic modulation influences tumor microenvironments. Clinical translation will further require systematic evaluation of safety, repeatability, and long-term effects, particularly as US-based BBB opening technologies are already being tested in patients.

## CONCLUSION

The growing body of research on the glymphatic system has fundamentally reshaped our understanding of how the brain regulates fluid transport, metabolic waste removal, and therapeutic distribution. Although glymphatic dysfunction has been extensively examined in neurodegenerative diseases, its role in brain cancers - and its relevance to drug delivery - has remained comparatively underexplored. This review synthesizes emerging evidence showing that US, across a range of frequencies and exposure conditions, can actively augment glymphatic influx, enhance CSF–ISF exchange, and accelerate interstitial solute clearance. These effects collectively highlight US as a promising tool for overcoming key barriers to therapeutic penetration in brain tumors.

Across the studies reviewed here, US was shown to modulate glymphatic transport through complementary biophysical and molecular mechanisms: amplification of perivascular pulsatility, vessel wall micron-scale deformation, TRPV4–CaM–AQP4–mediated fluid regulation, and microbubble-enhanced perivascular streaming. Importantly, these effects were demonstrated using diverse imaging modalities - including MRI, optical clearing–based 3D microscopy, and real-time two-photon imaging - providing converging evidence that US can influence multiple stages of glymphatic flow, from periarterial influx to parenchymal transport and perivenous efflux.

Overall, the studies reviewed here suggest that US has the potential to reshape therapeutic strategies for brain tumors by unlocking an underutilized fluid pathway - the glymphatic system - for targeted drug delivery. By integrating mechanistic insight with technological innovation, US-based glymphatic modulation may ultimately complement or transform existing treatments, offering new opportunities for noninvasive and precision medicine in neuro-oncology.

## DECLARATIONS

### AI and AI-assisted tools statement

During the preparation of this manuscript, the AI tool ChatGPT (version 5.3, released 2025-12-01) was used solely for language editing and FigureLabs (accessed 2026-02-05) was used to for generating the graphical abstract. These tools did not influence the scientific content of the work, including interpretation or conclusions. All authors take full responsibility for the accuracy, integrity, and final content of the manuscript.

### Copyright

© The Author(s) 2026.
